# Immunomodulation of Murine Chronic DSS-Induced Colitis by Tuftsin–Phosphorylcholine

**DOI:** 10.3390/jcm9010065

**Published:** 2019-12-26

**Authors:** Dana Ben-Ami Shor, Jordan Lachnish, Tomer Bashi, Shani Dahan, Asaf Shemer, Yahel Segal, Ora Shovman, Gilad Halpert, Alexander Volkov, Iris Barshack, Howard Amital, Miri Blank, Yehuda Shoenfeld

**Affiliations:** 1 Zabludowicz Center for Autoimmune Diseases, Sheba Medical Center Tel-Hashomer, Sackler Faculty of Medicine, Tel-Aviv University, Tel-Aviv 52620, Israel; jordanlac@gmail.com (J.L.); tomerbashi@gmail.com (T.B.); shish.dahan@gmail.com (S.D.); ShemerAsafMD@gmail.com (A.S.); segal.yahel@gmail.com (Y.S.); orashovman@walla.co.il (O.S.); tghalpert@gmail.com (G.H.); Howard.Amital@sheba.health.gov.il (H.A.); Miri.Blank@sheba.health.gov.il (M.B.); Yehuda.Shoenfeld@sheba.health.gov.il (Y.S.); 2 Department of Gastroenterology, Tel-Aviv Medical Center, Sackler Faculty of Medicine, Tel-Aviv University, Tel-Aviv 6423906, Israel; 3 Institute of Pathology, Sheba Medical Center Tel Hashomer, Sackler Faculty of Medicine, Tel-Aviv University, Tel-Aviv 52620, Israel; Alexander.Volkov@sheba.health.gov.il (A.V.); barshack@sheba.health.gov.il (I.B.)

**Keywords:** inflammatory bowel disease, colitis, phosphorylcholine, tuftsin

## Abstract

Helminths or their products can immunomodulate the host immune system, and this phenomenon may be applied as the basis of new anti-inflammatory treatments. Previously, we have shown the efficacy of tuftsin–phosphorylcholine (TPC), based on a helminth product, in four animal models of autoimmune diseases: arthritis, colitis, systemic lupus erythematosus, and experimental autoimmune encephalomyelitis. We demonstrated that TPC reduced inflammatory process ex vivo in peripheral blood lymphocytes (PBLs) and in biopsies from giant-cell arteritis. In the present study, we assessed the therapeutic potential of TPC treatment on a chronic colitis murine model. C57BL/6 mice with chronic colitis were treated with TPC after the third cycle of 2% dextran sodium sulfate (DSS). Oral TPC treatment resulted in amelioration of the colitis clinical manifestations exemplified by reduced disease activity index (DAI) score, expansion of mesenteric lymph nodes (MLN) T regulatory cells (shown by Fluorescence Activated Cell Sorting (FACS)), significant reduction in the expression of pro-inflammatory cytokines (IL-1β, IL17, IL-6, TNFα), and elevation in the expression of anti-inflammatory cytokine IL-10 (shown by RT-PCR). This study demonstrated the potential immunomodulatory effects of oral administration of TPC in a chronic colitis murine model. Further clinical trials are needed in order to evaluate this novel approach for the treatment of patients with inflammatory bowel disease.

## 1. Introduction

Inflammatory bowel disease (IBD) comprises two main clinical entities with both distinct and overlapping symptoms, ulcerative colitis (UC) and Crohn’s disease (CD). The etiology of UC and CD is yet to be completely understood, but is likely multifactorial. The currently held paradigm involves a complex interaction of three elements: genetic susceptibility, host immunity, and environmental factors [1,2]. UC and CD have been associated with a higher socioeconomic status, presumably due to relative underexposure to diverse environmental antigens during childhood—the hygiene hypothesis as it relates to intestinal mucosal immunity in IBD [3]. Joel Weinstock was the first to propose that the “deworming” of the developed world might be driving a rise in the prevalence of autoimmune disease [4]. The hypothesis has since been extended, as a strong correlation has been reported between high prevalence of parasitic worms (helminths) in certain geographic areas and immune protection from atopic, autoimmune, and auto-inflammatory diseases, including IBD [4,5,6,7,8]. 

A safe and effective therapy for IBD patients is still needed. First-line pharmacotherapy consists of 5-aminosalicylic acids and/or corticosteroids for acute episodes. Amino salicylates and thiopurines, but not corticosteroids, should be used as maintenance therapy. Intravenous corticosteroids, calcineurin inhibitors, and tumor necrosis factor-α antagonists may be indicated in severe, extensive, and refractory cases, but their effects wear off over time and adverse events can be limiting [9,10,11].

Helminths and their ova, employed in experimental animal models, have been shown to limit inflammatory activity in a variety of immune-modulated diseases [12,13,14,15]. This strong immunomodulatory activity is thought to be mediated by molecules secreted by the helminths to their surrounding environment. These products are collectively named excretory–secretory products, mainly comprising of phosphorylcholine (PC) moiety glycoproteins [16,17]. The immunomodulatory function of the helminth secretory products is attributed to PC. TPC (tuftsin–phosphorylcholine) is a small synthetic molecule based on helminth-derived products, composed of tuftsin–phosphorylcholine conjugate. The PC is a non-immunogenic, natural small molecule, while tuftsin is a physiological tetrapeptide (Thr–Lys–Pro–Arg) fraction of the IgG heavy chain molecule produced by enzymatic cleavage of the Fc domain of the IgG heavy chain in the spleen [18,19]. Tuftsin stimulates phagocytosis and has other stimulatory effects, including increased migration and activation of macrophages and enhanced chemotaxis of monocytes [18,19,20,21]. It has been used also as a natural adjuvant in the development of an influenza vaccine without side effects [18,19,22].

Previously, we showed that TPC delays glomerulonephritis onset in lupus-prone mice [23], moderates the destruction of joints in mice with collagen-induced arthritis (CIA) [24,25], and prevents colitis in DSS-induced murine acute colitis [26]. It also ameliorates development of experimental autoimmune encephalomyelitis, in a murine model of multiple sclerosis [27]. Ex vivo, TPC reduces the secretion of inflammatory cytokines by PBLs and by biopsies from patients with giant-cell arteritis [28].

Our experimental data represent the dawn of a promising new pharmacological field translating helminth derivatives into clinical therapies, and herein, we aimed to evaluate the effect of TPC treatment in a murine chronic colitis model.

## 2. Materials and Methods

### 2.1. Tuftsin–Phosphorylcholine (TPC) Synthesis

TPC was supplied by TPCera LTD. Jerusalem, Israel. TPC was diluted in commercial phosphate-buffered saline (PBS) (Biological Industries Israel Beit-Haemek Ltd, Kibbutz Beit-Haemek, Israel).

### 2.2. Mice 

Forty C57BL/6 male mice weighting 22–26 g (Envigo, Israel) were kept in a specific pathogen-free (SPF) environment in ventilated cages at the Sheba Medical Center (Israel) animal house. All experiments were permitted by the ethics committee of the Israeli Ministry of Health (no.696/11 and 1008/16).

### 2.3. Induction of Chronic Colitis and Protocol of Treatment

**Figure 1:**
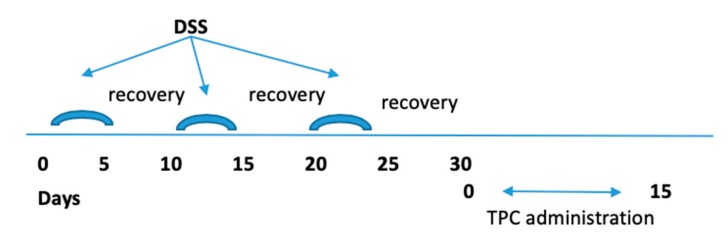
Chronic experimental colitis model and Tuftsin–Phosphorylcholine (TPC) administration.

Experimental colitis was induced in C57BL/6 male mice, 7 weeks old, by administration of 2% (w/v) dextran sulfate sodium salt (DSS) MW 36,000–50,000 Da, (MP Biomedicals Eschwege, Germany) in tap water, as previously described by us [26]. Chronic experimental colitis was induced by exposure to three cycles of: 2% DSS for 5 days, followed by a 5 day recovery period, as represented in Figure 1. To evaluate the severity of colitis, body weight, rectal bleeding, and stool consistency were observed to generate a disease activity index (DAI) score. After the last DSS cycle, mice were given a daily oral administration of TPC (50 μg/0.1 ml PBS), using a feeding tube, for the next 15 days. All mice were sacrificed at Day 45 after DSS administration. The colon was removed, and its length was measured. A fragment of the distal colon was fixed in 4% phosphate-buffered formalin for subsequent histological analysis. The mesenteric lymph nodes (MLN) were collected for further immunocyte isolation and RNA extraction.

### 2.4. Monitoring of DSS Chronic Colitis 

Weight loss, rectal bleeding, stool consistency, and survival were monitored daily. Change in body weight was defined as the percentage loss from the baseline weight (weight on the first day of treatment initiation). We used the Hemoccult test (SENSA, Beckman Coulter, USA) for occult bleeding detection, whereas findings of rectal bleeding indicated overt bleeds. Disease severity was assessed using a disease activity index (DAI). DAI was calculated by grading the following parameters on a scale of 0 to 4: intestinal bleeding (0, negative; 2, hemoccult; 4, gross bleeding), weight change (0, ≤1%; 1, 1–5%; 2, 5–10%; 3, 10–20%; and 4, ˃20%), and stool consistency (0, normal; 2, loose stools; 4, diarrhea). Mice were sacrificed after 15 days of treatment. Colon specimens were collected and gross colon shortening as well as histological changes were evaluated [26,29].

### 2.5. Histopathological Analysis

Distal colonic segments were resected and fixed in 4% formalin. We dehydrated tissue segments in alcohol (1 hour each in 70%, 80%, 90%, and 100%), xylene (three steps, 1 hour each), and then embedded them in paraffin. Following that, we sliced the paraffin blocks into 7 μm sections and stained them with hematoxylin–eosin (H&E) for histological microscopic assessment. Two blinded expert pathologists examined the slides and evaluated the severity of inflammation in view of histological features including loss of epithelium, crypt damage, depletion of goblet cells, and infiltration of inflammatory cells. 

### 2.6. T Regulatory Analysis by Flow Cytometry

Isolated MLN cells were incubated with anti-CD4-FITC and anti-CD25-APC. For intracellular staining of FOXP3, the cells were preincubated with a fixation solution, washed, resuspended in permeabilization solution (Serotec, Oxford, UK), and stained for FOXP3 (all from eBioscience, San-Diego, USA). The CD4^+^CD25^+^FOXP3^+^ T regulatory cells (Tregs) were analyzed by Fluorescence Activated Cell Sorting (FACS), with forward and side scatter gates adjusted to include all cells and to exclude debris (Becton Dickinson, Franklin Lakes, NY, USA). The gating was on the CD4^+^ T cells.

### 2.7. Quantitative Real-Time PCR

Total MLN RNA was isolated using a Total RNA Purification Plus Kit (Norgen Biotek, Canada) according to the manufacturer’s instructions. RNA concentration was measured using Nanodrop (Thermo Scientific). One μg total RNA was transcribed into cDNA with a High Capacity cDNA Reverse Transcription Kit (Invitrogen™, Carsbald, CA, USA) according to the manufacturer’s instructions. Gene expression was measured via real-time PCR performed on a StepOnePlus™ Real-Time PCR System (Applied Biosystems, Foster City, CA, USA) according to the manufacturer’s instructions.

Primer sequences (forward and reverse, respectively) were: IL-1β: 5′-GGA TGA GGA CAT GAG CAG CAC ATT C-3′; 3′- GGA AGA CAG GCT TGT GCT CTG A-5′; IL-17: 5′CCT CAA AGC TCA GCG TGT CC-3′; 3′-GAG CTC ACT TTT GCG CCA AG-5′; IL-6: 5′-ATG CTC CCT GAA TGA TCA CC-3′; 3′ TTC TTTGCA AAC AG CACA GC-5′; TNFα: 5′-AAG CCT GTA GCC CAC GTC GTA-3′; 3′-GGC ACC ACT AGT TGG TTG TCT TTG-5′; β-Actin: 5′-GAA ATC GTG CGT GAC ATA AAA G-3′; 3′-TGT AGT TTC ATG GAT GC CACA G-5′

The studied genes were normalized by the expression of β-Actin. The results are expressed as relative expression levels for each gene.

### 2.8. Statistical Analysis

Data are reported as a mean. We used repeated measures ANOVA followed by Tukey’s method, a single-step multiple comparison method, to assess weight change and DAI score differences between control groups and TPC-treated group. Kruskal–Wallis non-parametric analysis followed by Mann–Whitney test were used to test differences in stool consistency and intestinal bleeding between groups. We chose one-way ANOVA followed by Student’s t-test to analyze differences in cytokine responses and colon length between groups. *P*-values of less than 0.05 were considered statistically significant.

## 3. Results

### 3.1. TPC Significantly Attenuated Disease Activity in a Murine Chronic Colitis Model

**Figure 2:**
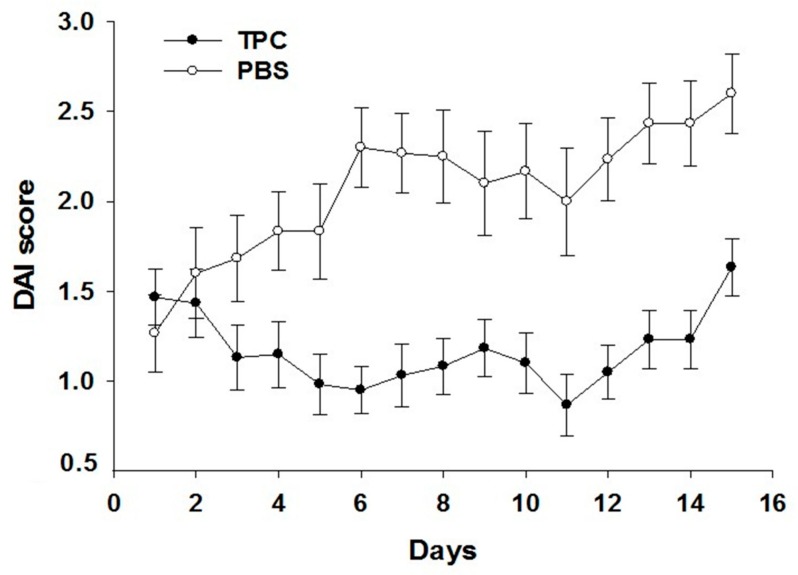
The effect of TPC on disease activity in a chronic colitis model. Day 0 in this graph presents day 30 of chronic colitis. DAI score: The levels of DAI score are presented as an average number, in each group of mice ±standard error (n = 20 per group), treated with tuftsin–phosphorylcholine (TPC) or phosphate-buffered saline (PBS). DSS mice that were treated with TPC had significantly lower average DAI score in comparison to mice treated with PBS (*p* = 0.001).

DSS-induced colitis is a commonly used model for studying various aspects of IBD, such as immune mechanisms, pathogenesis, genetic predisposition, and the role of microflora in disease pathogenesis. Following fifteen days of treatment, the disease severity measured by the DAI score was significantly higher in the mice treated with PBS (maximal DAI score of 2.6) compared to mice treated with TPC (maximal DAI score of 1.6) as depicted in Figure 2 (*p* < 0.001).

When comparing the TPC and PBS groups, it was evident that the PBS group presented with significantly more intestinal bleeding than the TPC group (*p* < 0.001). When examining the mice on Day 15, we found 13 mice in the PBS-treated group that suffered from overt rectal bleeding (77%) and 4 mice with positive occult bleeding (23%), while in the TPC-treated mice, only 7 mice experienced overt rectal bleeding (35%), 11 mice had occult blood (55%), and in 2 mice no form of bleeding was observed (10%). The average bleeding scores were 3.6 and 2.5 in the PBS- and TPC-treated groups, respectively.

We found that oral treatment with TPC significantly reduced diarrhea (*p* < 0.001). At the end of treatment, eight mice (47%) of the control group presented with diarrhea and nine mice (53%) had a loose stool consistency. The average stool consistency score in the PBS-treated group was 3.1.

In the TPC-treated group, only 3 mice had diarrhea, 15 mice (75%) had a loose stool consistency, and 2 mice (10%) displayed normal stool consistency. The average stool consistency score in the TPC-treated group was 2.1.

**Figure 3:**
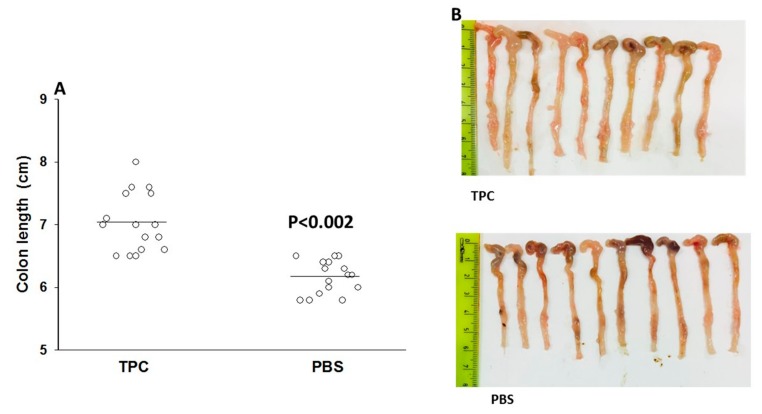
The effect of tuftsin–phosphorylcholine (TPC) on colon length. Colon length measurements represented in centimeters, on Day 45 of experiment, after three cycles of 2% DSS and 15 days of treatment with TPC or phosphate-buffered saline (PBS). (**A**) TPC-treated mice demonstrated significantly less shortening of the colon (*p* < 0.002). (**B**) Comparison of the colon length between TPC treated mice and PBS subjected mice.

Measurements of the colon were evaluated after sacrificing the mice. The colons of the control PBS-treated mice had an average length of 6.1 cm and were significantly (*p* < 0.002) shorter than the colons of the TPC-treated mice, which had an average length of 7 cm (Figure 3).

Finally, the applied DSS regimen led to 15% (3/20) mortality in untreated mice compared to complete survival of TPC-treated mice.

### 3.2. TPC Reduced Inflammation in Murine Chronic Colitis Model

**Figure 4:**
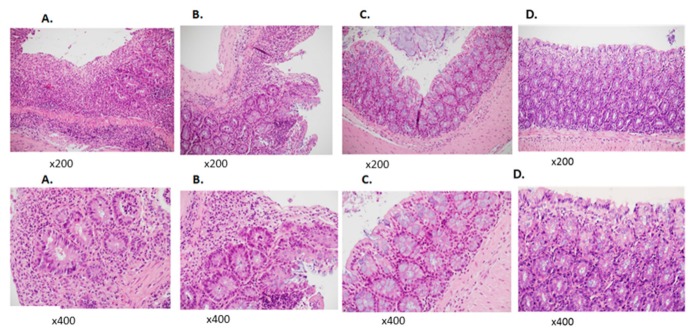
Histological analysis of distal colon sections in chronic colitis mice. Histological analysis: H&E staining in representative distal colon section from each studied group of chronic colitis mice magnification of ×200 and ×400. (**A**) PBS control mice: massive infiltration of lymphocytes, destruction of the normal structure, and cryptitis. Histological score for DSS = 12 points (over 10% loss of epithelium = 3 points, over 20% loss of crypts = 3 points, severe depletion of goblet cells = 3 points, severe infiltration of inflammatory cells = 3 points). (**B**) TPC-treated mice: mild pathology of cryptitis. Histological score for DSS = 6 points (over 10% loss of epithelium = 3 points, 10–20% loss of crypts = 2 points, moderate depletion of goblet cells = 2 points, mild infiltration of inflammatory cells = 1 points). (**C**) TPC-treated mice: No significant infiltration of lymphocytes with no cryptitis. Histological score for DSS = 0 points (no loss of epithelium = 0 points, no loss of crypts = 0 points, no depletion of goblet cells = 0 points, no infiltration of inflammatory cells = 0 points). (**D**) Normal healthy column.

Microscopic assessment of colonic damage was performed 45 days after colitis induction. PBS-treated mice demonstrated a severe chronic active inflammation involving the mucosal layer, with a severe disruption of the normal architecture, massive infiltration of lymphocytes, cryptitis, and crypt loss (Figure 4A). PBS control mice: massive infiltration of lymphocytes, destruction of the normal structure and cryptitis. Histological score for DSS = 12 points (over 10% loss of epithelium = 3 points, over 20% loss of crypts = 3 points, severe depletion of goblet cells = 3 points, severe infiltration of inflammatory cells = 3 points).

Histological assessment of colon sections upon treatment with TPC revealed a better microscopic outcome. Overall, 30% exhibited severe inflammation similarly to the PBS-treated mice. Another 40% had mild to moderate inflammation, with increased lymphocyte infiltration and mild cryptitis (Figure 4B). Figure 4B illustrates a mild cryptitis pathology. Histological score for DSS = 6 points (over 10% loss of epithelium = 3 points, 10–20% loss of crypts = 2 points, moderate depletion of goblet cells = 2 points, mild infiltration of inflammatory cells = 1 point).

As illustrated in Figure 4C, 30% of the mice treated with TPC maintained a conserved colon structure with only minimal lymphocyte infiltration. No significant infiltration of lymphocytes and no cryptitis was observed. Histological score for DSS = 0 points (no loss of epithelium = 0 points, no loss of crypts = 0 points, no depletion of goblet cells = 0 points, no infiltration of inflammatory cells = 0 points).

### 3.3. MLN T Regulatory Cell Expansion upon TPC Treatment in the Murine Chronic Colitis Model

**Figure 5:**
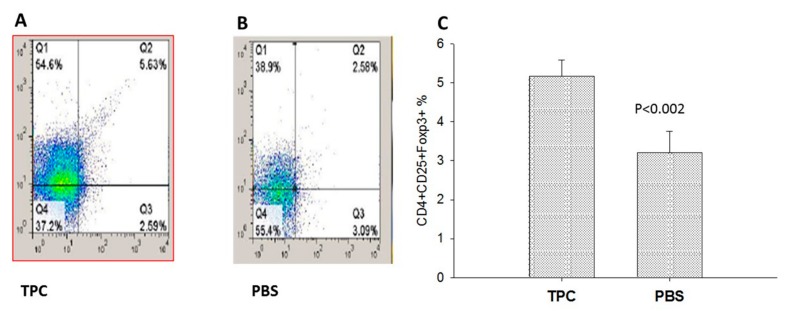
T regulatory cells expansion in isolated mesenteric lymph nodes of TPC-treated chronic colitis mice. (**A**). The data is presented as a percentage of Tregs CD4^+^CD25^+^FOXP3^+^ expansion in isolated mesenteric lymph nodes of TPC and PBS-treated mice (n = 20). Values are the mean ± SD, p < 0.0001. (**B**) Representative flow cytometry analyses of Tregs CD4^+^CD25^+^FOXP3^+^ (gated on CD4^+^) in mesenteric lymph nodes derived from the TPC and PBS-treated mice. TPC—5.63% PBS—2.58%. (**C**) Percentage of T regulatory upon treatment with TPC in comparison to treatment with PBS.

To evaluate the effect of TPC on the MLN T regulatory cell numbers, MLN cells were isolated and the CD4 T cells were tested for CD4^+^CD25^+^FOXP3^+^ T regulatory (Treg) cells by FACS. As illustrated in Figure 5A–C, TPC enhanced expansion of Treg cells from 2.58% in the PBS-treated group (n = 20) and to 5.6% in the TPC-treated group (n = 20), *p* < 0.002. The cells were gated on CD4^+^ T cells.

### 3.4. TPC Immunomodulated MLN Cytokine mRNA Levels in Murine Chronic Colitis Model

**Figure 6:**
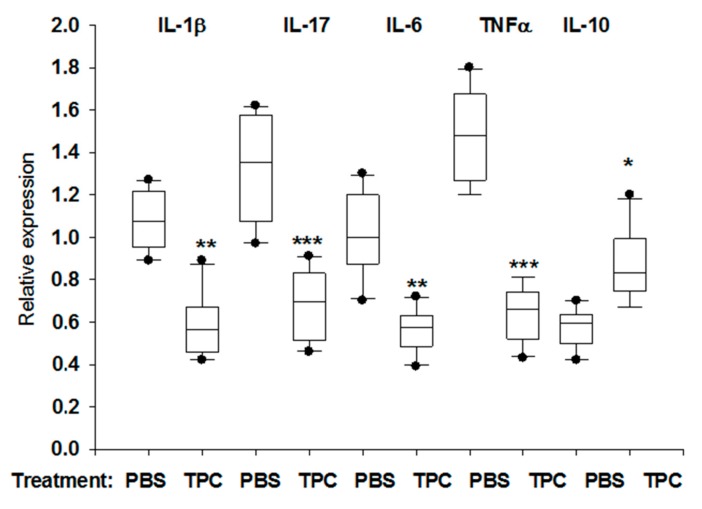
Cytokines gene expression of IL-1β, IL-17, IL-6, TNFα, IL-10 by mesenteric lymph nodes (MLN) from oral administration of tuftsin–phosphorylcholine (TPC) in comparison to phosphate-buffered saline (PBS) subjected mice. The data was normalized with β-Actin expression. One Asterix *p* < 0.001, two Asterix *p* < 0.02-0.03, three Asterix *p* < 0.001.

At the end of the experiment, the effect of TPC treatment on pro- and anti-inflammatory cytokine mRNA expression levels was analyzed. As shown in Figure 6, we compared the expression of IL-1β, IL-17, IL-6, and TNFα MLN mRNA transcripts to the anti-inflammatory IL-10 mRNA transcripts. There was a significant reduction in the mRNA transcripts of pro-inflammatory cytokines IL-1β (1.08 ± 0.58, *p* < 0.003), IL-17 (1.32 ± 0.24, *p* < 0.001), IL-6 (1.01 ± 0.19, *p* < 0.02), and TNFα (1.48 ± 0.21, *p* < 0.001). Meanwhile, mRNA expression of IL-10 by MLN was elevated upon treatment with TPC (0.57 ± 0.87, *p* < 0.009). The data show a significant difference on inhibition of mRNA transcripts of pro-inflammatory cytokines and enhanced expression of mRNA transcripts of IL-10 anti-inflammatory cytokine.

## 4. Discussion

This study demonstrated the immunomodulatory effect of TPC, a small molecule (~1 KD) based on a helminth product, in a murine model of chronic colitis. Oral TPC, initiated after chronic DSS-induced colitis was established, resulted in a substantial improvement in all observed clinical outcomes and histological manifestations. TPC significantly promoted the CD4^+^CD25^+^FOXP3^+^Treg phenotype expansion in MLN. Furthermore, TPC significantly inhibited the expression of pro-inflammatory cytokines IL-1β, IL-17, IL-6, and TNFα mRNA transcripts, whereas mRNA transcription of IL-10 was enhanced. It is noteworthy that CD4^+^CD25^+^ Treg has been shown to prevent and resolve murine colitis, and the cure of murine colitis by these cells was dependent on the presence of IL-10 [30,31]. Moreover, although it was found that there is expansion of Tregs in both MLN and the lamina propia of inflamed intestinal areas of UC patients, their suppressive activity may be abrogated or they are unable to counterbalance the chronic mucosal inflammation process [32]. Therefore, our findings demonstrate the clinical importance of increasing MLN Treg numbers and IL-10 expression upon oral TPC treatment, in parallel to its significant effect on clinical symptoms and histopathology in chronic inflammatory colitis.

The current results support our previous studies on the immunomodulatory activities of TPC, exemplified by inhibition of pro-inflammatory cytokines and enhancement of anti-inflammatory IL-10 levels, associated with elevated expansion of Treg cells and Breg cells [23,24,25,26,27]. In the past, we proved the bifunctional in vitro activity of TPC [25]. In order to explain the increased expression of IL-10 and the increased number of Tregs in the MLN cells, we claim that it is related to the bifunctional activity of TPC [25]. In a previous study, we showed that (a) the phosphorylcholine end of the molecule binds TLR4 and inhibits NFkB activity, whereas (b) tuftsin binds macrophages and Treg through the interaction of arginine with the neuropilin-1 receptor. TPC causes a shift from M1 pro-inflammatory macrophages to M2 anti-inflammatory macrophages secreting IL-10 [25].

Prior results from our group confirmed the importance of IL-10 in the inflammatory suppressive effect of TPC [25,26]. Our finding is supported by additional studies showing that increased IL-10 production occurs in parasite-mediated amelioration of autoimmune diseases [33,34,35,36,37]. Another potential player in the immune properties of TPC is IL-1β. Mucosal barrier breach induced by IL-1β has been proposed as a trigger of mucositis onset. Studies in mice suggest that epithelial tight junction dysfunction and mucositis significantly improve with antibody neutralization of IL-1β [38,39]. Previously, we observed a decrease in colonic IL-1β levels following TPC treatment [26].

In the past few decades, investigators have largely focused on the use of helminths and their secreted products as therapeutic interventions in disease treatment [40]. Helminths cause changes in the immune systems of their hosts, including an altered immunological response to antigens. This effect has implications for the treatment of IBD, as well as other diseases caused by immune dysregulation [41]. The use of helminths as a therapeutic intervention is a novel concept in disease management. In spite of its novelty, the concept is already at an advanced experimental stage for several diseases through randomized controlled trials (RCTs) [42,43,44,45,46,47,48,49]. However, there is still insufficient evidence to allow firm conclusions on the safety and efficacy of helminth therapy in IBD [40].

In conclusion, TPC as a bifunctional molecule based on a helminth product, prevented severe colitis in DSS-induced acute colitis model and had a significant clinical and histological therapeutic effect in the same DSS-induced chronic colitis model. Our data represent an experimental proof-of concept that TPC therapeutic efficacy is associated with prevention of murine colitis development [26], as well as with treatment of chronic murine colitis. This has important implications in suggesting TPC as a synthetic small molecule based on helminth-derived products for treating colitis in humans. Although chemically induced colitis is a well-established, valuable tool in testing therapeutic strategies in the preclinical stage, it does not fully mimic the immune phenomena of IBD in humans; therefore, further research is needed.
